# Adrenalectomy for solitary adrenal metastasis from colorectal cancer: A case report

**DOI:** 10.1186/1757-1626-1-49

**Published:** 2008-07-18

**Authors:** Christopher Kosmidis, Christopher Efthimiadis, George Anthimidis, Sofia Levva, Georgia Ioannidou, Thomas Zaramboukas, Christos Emmanouilides, Sofia Baka, Maria Kosmidou, Georgios Basdanis, Epaminondas Fachantidis

**Affiliations:** 1 Departments of Surgery, Interbalkan European Medical Center, Thessaloniki, Greece; 2 Departments of Radiology, "Panagia" General Hospital, Thessaloniki, Greece; 3 Departments of Pathology, Interbalkan European Medical Center, Thessaloniki, Greece; 4 Departments of Oncology, Interbalkan European Medical Center, Thessaloniki, Greece; 5 Departments of Internal Medicine, Interbalkan European Medical Center, Thessaloniki, Greece

## Abstract

**Background:**

Patients with adrenal metastasis from various primary tumours are regarded as cases of diffuse systemic spread and considered unsuitable for surgical resection. We herein report an operable case of heterochronic adrenal metastasis from colorectal carcinoma in a 63-year-old woman.

**Case presentation:**

Sixteen months after low anterior resection for the primary tumour, left lower pneumonectomy was performed for a solitary lung metastasis. Four months later a right adrenal metastasis was detected by magnetic resonance imaging (MRI), as sole evidence of metastatic disease. A right adrenalectomy was performed. The histopathological examination revealed adenocarcinoma compatible with the colorectal carcinoma resected 19 months earlier. The patient received adjuvant chemotherapy after each operation and is alive and free of disease 21 months after the adrenalectomy.

**Conclusion:**

The possibility of adrenal metastasis should be considered in the follow-up of patients after primary surgery for colorectal cancer, even though other sites are the main metastatic sites. Although the prognosis of adrenal metastasis from colorectal cancer is poor, we suggest that patients with solitary adrenal metastasis may benefit from complete removal of it.

## Introduction

Adrenal metastasis most commonly occurs in patients with lung, breast and renal cancer [1,2]. In general, metastasis to the adrenal gland is regarded as an indicator of widespread disease, but in rare cases, isolated adrenal metastasis can be found [3-6]. Although the incidence varies among reports, it is generally accepted that adrenal metastasis from colorectal cancer (CRC) is relatively rare. As a result of recent advances in radiological noninvasive techniques, metastasis to the adrenal gland can be detected during follow up after primary operations for CRC. Whether isolated metastasis to the adrenal gland needs to be resected, remains controversial. Surgical resection of the involved adrenal gland seems to be able to provide a survival benefit for selected patients, according to several previously reported cases, whereas radiation and chemotherapy have relatively poor results for these lesions [1,4,7].

## Case presentation

A 63-year-old-woman presented with lower abdominal pain and anal bleeding on defecation. Colonoscopy revealed intraluminal stenosis of the colorectal junction and biopsy specimens were obtained. Histologically, a well-differentiated adenocarcinoma was diagnosed. There was no evidence of metastasis, based on the results of abdominal computed tomography (CT) and chest X-ray, while (carcinoembryonic antigen) CEA was normal at 2.36 ng/ml. The patient underwent low anterior resection for CRC on 14 July 2004. The lesion was a stage Â2 (Astler-Coller) tumour, 3,0 × 2.5 cm in size, located in the rectosigmoid colon. Microscopic examination showed well-differentiated adenocarcinoma, penetrating the muscularis propria, without lymphatic and vascular invasion and without metastases to 15 dissected lymph nodes. According to the classification of TNM (tumour, lymph nodes, metastasis), the disease was stage II. Her postoperative course was uneventful, and she was started on adjuvant chemotherapy, comprised of oxaliplatin i.v., 85 mg/m^2 ^(on day 1), leukovorin i.v., 200 mg/m^2 ^(on days 1,2), 5 FU (fluorouracil) i.v., bolus 400 mg/m^2 ^(on days 1,2), 5 FU i.v., 22 hours-infusion, 600 mg/m^2 ^(on days 1,2), every 2 weeks for 12 cycles.

**Figure 1:**
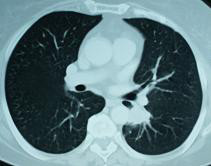
Abdominal C. T. showed a tumour 3 cm in diameter in the lower lobe of the left lung.

**Figure 2:**
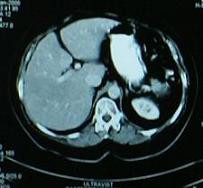
A CT scan showed a right adrenal mass, without evident signs of metastases to other organs.

**Figure 3:**
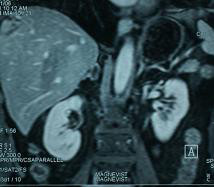
Magnetic resonance imaging showing a nodule in the right adrenal gland.

Four months after the operation the patient underwent a cholecystectomy for acute cholecystitis, confirmed by ultrasound. Microscopic examination showed no evidence of malignancy of the gallbladder. One year after the cholecystectomy, a follow up abdominal C.T. detected a tumour 3 cm in diameter in the lower lobe of the left lung (Figure 1). The level of serum CEA remained within the normal range. Colonoscopy, mammography, and scintiscan (a two-dimensional record of the distribution of a bone-seeking radioactive material, obtained by means of a scanning scintillation counter) of the bones showed no signs of metastasis. Left lower pneumonectomy was performed; the tumour's maximum diameter was 3 cm and microscopic examination showed complete removal of a metastatic, moderately differentiated adenocarcinoma, without vascular infiltration, compatible with the resected rectosigmoid carcinoma. Immunohistochemically the tumour cells were strongly positive for Keratin 20 and negative for Keratin 7 and (thyroid transcription factor 1) TTF-1 antigen. However, one of the 13 resected peribroncheal lymph nodes was involved. The chemotherapy was changed to bevacizumab i.v. 5 mg/kg (on day 1), irinotecan i.v., 180 mg/m^2 ^(on day 1), leukovorin i.v., 200 mg/m^2 ^(on days 1,2), 5 FU i.v., bolus, 400 mg/m^2 ^(on days 1,2), 5 FU i.v., 22 hours-infusion, 600 mg/m^2 ^(on days 1,2), every 2 weeks for 6 cycles. Four months after the lower pneumonectomy a follow-up CT-MRI identified a nodule in the right adrenal gland, 2 × 1 cm in size [4]. Since there were no other signs of local recurrence or distant metastasis on radiological, endoscopic and laboratory examinations, with CEA level still within the normal range, the right adrenal mass was regarded as an isolated heterochronic metastasis from CRC and resection was considered. Right adrenalectomy through a midline abdominal approach was performed. The tumour's maximal diameter was 2.1 cm while the maximum diameter of the adrenal gland was 5.2 cm (Figures 2 &3). Histopathological examination and immunohistochemical studies showed complete removal of a well-differentiated adenocarcinoma compatible with the rectosigmoid carcinoma resected one year and 7 months earlier. The chemotherapy was once again changed to cetuximab i.v., 250 mg/m^2^, irinotecan i.v., 125 mg/m^2 ^(on days 1,8), every 3 weeks for 6 cycles.

The patient is alive and free of disease 3 years and 4 months after the primary operation for CRC and 21 months after the right adrenalectomy.

## Discussion

The incidence of adrenal metastasis from CRC ranges from 0.15% to 17.4% with a mean percentage of 16%, according to different reports [1]. By performing a medline literature search, we were able to identify 24 previously reported cases of resection of adrenal metastasis from CRC [1-9]. Five of these, including ours, had also lung metastasis either synchronous or metachronous [1-9]. It is conceived that a number of routes of adrenal metastasis from CRC exist, including systemic venous, portal venous, arterial, and lymphatic routes [2]. Katayama et al. suggested that there is a route of hematogenous metastasis from the primary lesion via the lung to the adrenal gland [3]. Thus, the significance of long-term follow-up after resection of CRC for early detection of adrenal metastasis, especially after resection of lung metastasis, should not be overlooked.

Patients' serum CEA levels are usually elevated when a metastasis is diagnosed, and CEA is considered to be useful for indicating the presence of adrenal metastasis after surgery for CRC [3]. However, the serum level of CEA in our patient was normal despite the occurrence of lung and adrenal metastasis. Nevertheless, the ability to detect clinically silent adrenal metastasis has increased due to the widespread use of imaging modalities, including ultrasonography, CT and MRI. Therefore, in addition to measuring serum CEA, these modalities can be used in the follow up of patients who undergo a primary operation for CRC.

Adrenal metastasis from CRC is usually a part of systemic disease, accompanied by poor prognosis and has been regarded as having no indication for surgical resection [3,4]. On the other hand, resection of solitary adrenal metastasis from CRC should be offered to patients with significant (> 6 months) disease free interval, since it may improve chances of survival [1,3]. However, patients whose adrenal metastases are discovered synchronously or within 6 months of diagnosis of the primary tumour are less amenable to cure from metastasectomy. This may be due to the intrinsic biologic aggressiveness of the tumour and its metastases. Kim et al suggest that all metastases are present microscopically or macroscopically at the time of primary tumour diagnosis and that metastatic deposits which remain clinically undetectable beyond 6 months reflect the slow-growing nature of these tumours [1].

One could argue that it was the change in chemo regime rather than the surgical resection that helped the patient. In a recent review paper [10], Gundgaard et al reported median time to progression (mTTP) up to 7 months and a medium overall survival (mOS) of 16 months with third line chemotherapy. The highest mÔÔP, 9.8 months, was reported for the combination of cetuximab combined with irinotecan, the treatment our patient received.

It is difficult to determine whether adrenal metastasectomy has affected the natural history of our patient. However, it is evident that nonsurgical treatment of solitary adrenal metastases has been associated with poor survival. The small number of patients with isolated adrenal metastasis of colorectal carcinoma makes a randomized, prospective trial comparing surgical resection to other treatment modalities highly unlikely.

Laparoscopic adrenalectomy has rapidly replaced open adrenalectomy as the procedure of choice for benign adrenal tumours in the last decades. However, laparoscopic resection is controversial for large, potentially malignant adrenal tumours and necessitates experience in open surgery and advanced laparoscopic surgery [11]. Therefore it is essential to differentiate benign and non-functional lesions from malignant or hormonally active ones so that appropriate treatment strategies can be initiated. In our case, since the adrenal mass was possibly malignant, we performed an open adrenalectomy.

## Conclusion

Adrenal metastasis from CRC via the lung to the adrenal gland is considered to be relatively rare. It is generally accepted that a solitary adrenal metastasis from adenocarcinoma of the colon and rectum should be resected to achieve good prognosis. Therefore, it is important to consider the possibility of adrenal metastasis from CRC during follow-up after the primary operation. To detect adrenal metastasis early, radiological modalities such as US, CT and MRI as well as the measurement of serum CEA, should be done regularly. New modalities, such as (2-[18F] fluoro-2-deoxy-Dglucose (FDG) and positron emission tomography) 18F-FDG PET and (Computed Tomography-Positron Emission Tomography) CT-PET may offer better chances to detect clinically silent adrenal metastasis in the future.

## Consent

Written informed consent was obtained from the patient for publication of this case report and any accompanying images. A copy of the written consent is available for review by the Editor-in-Chief of the ≪Cases Journal≫.

## Competing interests

The authors declare that they have no competing interests.

## Authors' contributions

All authors contributed the same.
